# Cancer Multidisciplinary Team Meetings: Evidence, Challenges, and the Role of Clinical Decision Support Technology

**DOI:** 10.4061/2011/831605

**Published:** 2011-07-17

**Authors:** Vivek Patkar, Dionisio Acosta, Tim Davidson, Alison Jones, John Fox, Mohammad Keshtgar

**Affiliations:** ^1^Breast Unit, Royal Free Hospital, London NW3 2QG, UK; ^2^University College London, London W1W 7EJ, UK; ^3^Department of Engineering Science, Oxford University, Oxford OX1 3PJ, UK

## Abstract

Multidisciplinary team (MDT) model in cancer care was introduced and endorsed to ensure that care delivery is consistent with the best available evidence. Over the last few years, regular MDT meetings have become a standard practice in oncology and gained the status of the key decision-making forum for patient management. Despite the fact that cancer MDT meetings are well accepted by clinicians, concerns are raised over the paucity of good-quality evidence on their overall impact. There are also concerns over lack of the appropriate support for this important but overburdened decision-making platform. The growing acceptance by clinical community of the health information technology in recent years has created new opportunities and possibilities of using advanced clinical decision support (CDS) systems to realise full potential of cancer MDT meetings. In this paper, we present targeted summary of the available evidence on the impact of cancer MDT meetings, discuss the reported challenges, and explore the role that a CDS technology could play in addressing some of these challenges.

## 1. Background of MDT Meetings in Cancer Management

Multidisciplinary team meetings are also known as tumour boards, multidisciplinary cancer conferences, multidisciplinary case reviews, or multidisciplinary clinics, in different health care systems. These different terms may represent the variations in the organisational structure, membership, approach, focus, and the decision processes of these meetings [1]; however, they all provide a forum for multidisciplinary cancer teams to regularly convene and discuss the diagnostic and treatment aspects of patient care. 

 In MDT meetings the emphasis is on collaborative decision-making and the treatment planning, where the core team members of relevant specialties participate through the MDT meetings to share their knowledge and make collective evidence-based recommendations for patient management. The concept of a multidisciplinary forum to share knowledge among clinicians from different disciplines is not new to oncology. Tumour boards have existed in the United States for the last 50 years [2]. However, until recently, their primary goal was educational rather than improving patient care. In the 1980s a shift towards community-based cancer care in the United States provided a stimulus to the development of tumour boards, which facilitated sharing of information amongst participating physicians with positive benefits for quality of care [3]. In the last two decades there has been a transition of the primary goal of tumour boards from education to the delivery of care. In the United Kingdom, the overhaul of cancer services and subsequent rapid adoption of MDMs in the last decade has been primarily driven by political and public pressure resulting from a series of reports published in the late 1990s which highlighted wide variation in patients' care [4]. Many other European countries and Australia were quick to adopt the MDT model for cancer care [5, 6].

## 2. The Rationale behind Regular MDT Meetings

In the United Kingdom, the National Health Service (NHS) National Cancer Plan, published in 2000, endorsed the multidisciplinary team model for the management of cancer patients, and regular multidisciplinary team meetings have since been endorsed as the means to achieve this. The rationale for regular MDT meeting is multidimensional. These forums aim to ensure that all patients receive timely diagnosis and treatment, that patient management is evidence-based, and that there is continuity of care. The regular meetings facilitate information exchange and regular communication flow between all those involved in treatment of the patient. The team members can monitor adherence to evidence-based guidelines and can streamline the resources for improved management strategies, lower waiting times and enhanced cost effectiveness. The MDT meeting provides an opportunity for education and learning to its members and trainee doctors. They may also improve the well-being and work satisfaction of individual team members. Cancer MDT meetings are also viewed as an important opportunity to identify patients who are eligible for research trials. It naturally follows that if the team functioning, communication and decision-making are improved, then ultimately both patient care quality and patient outcomes will improve. 

In the following section, we examine the empirical evidence available in the literature to support the claimed benefits of cancer MDT meetings.

## 3. The Evidence on the Impact of Cancer MDT Meetings

We performed a detailed review of published evidence on the effectiveness of cancer MDT meetings. Table 1 summarises the available evidence on the effectiveness of cancer MDTs. As depicted in this table, the published empirical evidence to support the benefits of cancer MDT meetings is weak and limited. It is paradoxical that we expect our individual clinical decisions to be based on sound empirical evidence but not the same for organisational decisions. 

### 3.1. Studies Showing No or Negative Impact

We identified only one randomised controlled trial [7] that reported on survival. The intervention arm of the trial consisted of a two-stop centralised diagnostic pathway followed by MDT review while the control arm was a conventional pathway without MDT review. The patients recruited (*N* = 88) were those with suspected lung cancer, who were considered fit for a CT scan and tissue biopsy. The study reported no statistically significant difference in the quality of life (QOL) at six weeks and the survival at two years between intervention and control arm. Two other observational studies evaluating the role of MDT meetings in high-grade glioma [8] and nonsmall cell lung cancer [9] also reported no statistically significant difference in survival between the MDT and control groups. 

The study [20] evaluated the influence of lung cancer MDT meetings on the quality of decision-making and reported that the change in net utility loss brought about by multidisciplinary team discussion was not significantly different from zero, and team discussion did not improve the quality of decision-making overall.

### 3.2. Studies Showing Positive Impact

We identified eight observational studies in different cancer domains that reported improvement in various patient outcomes, attributed to the MDT. The study [10] compared the outcomes of oesophageal cancer patients in the period after introduction of specialist team and regular MDT meetings (1998–2003, *n* = 67) to those of an earlier period when no MDT meetings were held (1991–1997, *n* = 77). Authors reported lower operative mortality (5.7% versus 26 %, *P* = 0.004) and improved 5-year survival (52% versus 10%, *P* = 0.0001) in the MDT group. The Scottish study [12] reviewed outcomes of ovarian cancer patients treated in 1987 (*N* = 533) and found a survival difference between patients managed through a multidisciplinary clinic (MDC) and those not managed through an MDC (*P* < 0.001). A before and after study [11] reported a statistically significant but modest improvement of 3.2 months in median survival of patients with inoperable nonsmall cell lung cancer (3.4 versus 6.6 months, *P* < 0.001). A population based before-and-after study [13] compared the survival of patients with invasive cancers, from the Hoag Hospital tumor registry and reported significant improvement in relative 5 year survival (71% versus 63%, *P* < 0.001) in favour of MDT group. The Scottish study of elderly people with non-small cell lung cancer [14] reported improvement in survival following the introduction of MDT meetings and site specialisation.

The study of patient satisfaction [15] in newly diagnosed breast cancer patients before and after the establishment of multidisciplinary breast clinics, reported in favour of MDT group (*P* < 0.001). In an audit study, Burton et al. [22] compared preoperative MRI in consultation in MDT meeting to preoperative MRI without MDT consultation in rectal cancer patients. They reported that for the incidence of positive circumferential resection margin (CRM) was significantly higher in the group without MDT consultation (26% versus 1%). 

A study [17] found that a clinical trial recruitment rate in a population of 2,935 colorectal cancer patients diagnosed in year 2000, in twelve French administrative districts, accounting for 15% of the geographical area of France. They discovered that the patients who were managed through MDT, the trial recruitment rate was significantly higher (10.3%) compared to that in patients not managed through MDT (5.1%, *P* < 0.001). 

Stephens et al. reported an increase in the number of patients with oesophageal cancer being staged in the MDT group (100%) as compared to the historical control (54%, *P* = 0.001) [10]. A greater proportion of patients also received radical radiotherapy in the MDT group (5%) compared with that of the control (0.5%, *P* < 0.001). In another study in nonsmall cell lung cancer (NSCLC) patients [11] there was an increase in the proportion of patients receiving chemotherapy (23% in the MDT group versus 7% in control, *P* < 0.001) and the proportion of patients being staged. A study [16] reported a 30% increase in the annual lung cancer resection rate (from 14.7 to 19 resections per year) following the introduction of a telemedicine MDT meeting. Back et al. [8] retrospectively reviewed the patients referred to a large radiation therapy centre in Singapore between 2002 and 2006. They reported an increase in the proportion of patients receiving chemotherapy for high grade glioma (55% versus 17 %) and also an increase in the number of patients receiving postoperative imaging within 5 days of surgery in the former group (86% versus 59%) both in favour of MDT group. In a study comparing the staging accuracy of individual imaging modalities, endoscopic ultrasound (EUS), CT scan, and Laparoscopic ultrasound (LUS) for gastric and oesophageal cancer against that of collective MDT staging [18] found that collective MDT staging was more accurate compared with any individual imaging techniques. 

We identified only one study [19] that evaluated the cost effectiveness of MDC against a control group. They looked at cost effectiveness of multidisciplinary melanoma care at a large academic medical center in the United States compared to traditional community-based treatment. The authors concluded that multidisciplinary care would save $1600 per patient when compared with conventional care.

 We did not find any studies directly comparing impact of the MDT meetings on individual team member's mental health. A study of breast cancer teams [21] used the General Health Questionnaire GHQ-12 to assess psychiatric morbidity, compared it with historic control and concluded lower prevalence of psychiatric morbidity in breast cancer MDT members.

## 4. Evidence Summary

On balance, the number of published studies reporting positive impact of cancer MDTs is more than the studies that failed to show benefits. However, the design of almost all of the studies identified in this paper was poor. The studies often used before-and-after designs, which are considered as weak evidence for establishing causal associations, because of multiple potential confounders. The knowledge about cancer and available diagnostic and treatment options continuously evolves over time so better outcomes in later periods are more likely. Some of these studies used historical controls which are well known for introducing significant biases resulting in spurious results. In many of the before-and-after studies, no adjustment for any confounders were done and in some [10] a stage drift was found between the groups. A major problem that we observed in majority of these studies was multiple concurrent organisational changes in the intervention group, which might have accounted for the observed outcome benefits. For example, along with establishment of MDT meetings, other organisational changes like centralisation of process and increased caseload [7, 16], appointment of new specialists [9, 10], and streamlining of clinical pathways [15] made the interpretation of causal links between the MDT meetings and observed outcomes extremely difficult. The only randomised control trial that we found was a pilot RCT with modest sample size (88 patients in total) and very short follow-up period. The trial was not powered sufficiently so making interpretation of the results very difficult. 

Our paper findings are in line with other previously published reviews [1, 23–25], all highlighting the paucity of good-quality evidence to support use of the MDM in different tumour contexts. However, it is important not to interpret the absence of good-quality evidence as evidence of ineffectiveness. One of the main reasons for the paucity of the data is the practical difficulties in setting up conventional randomised controlled trials for a complex interventions like MDT meetings [26]. The reality is that cancer MDT meetings have already been established as a standard of care in many healthcare systems making new RCTs in future unlikely. 

## 5. Supporting Overburdened MDT Meetings to Realise Their Full Potential

It seems intuitively obvious that the intervention likes MDT meetings that are aimed at improving information exchange and regular communication flow between team members should benefit the overall care process. This is indirectly supported by the fact that MDTs are well accepted by the health community, despite the lack of robust evidence. As many health care systems have already invested and committed to the MDT model, the best way forward would be to focus on improving their conduct to obtain maximum leverage and to exploit the opportunity created by these meetings to gather data on patient and process measures to prospectively assess and document their performance and effectiveness.

 Our paper identified pragmatic challenges and shortcomings in the current conduct of cancer MDT meetings which are summarised in Table 2. A survey by Haward et al. of 72 breast multidisciplinary teams in the UK found wide variation in the treatments received [18] by specific patient subgroups. Furthermore, there are no formal mechanisms in place to evaluate compliance with best practice. In another postal survey of breast MDMs in the UK [27], 29% of respondents stressed the need for better preparation and 6% noted no recording of decisions made in the MDM, which raises concern over the decision from the meeting being available for the patient and any members not present. In the United Kingdom the national cancer peer review programme, launched in 2001, provides measurable standards to assess teams' adherence to the guidance. The analysis of data collected in the national cancer peer review programme including over 1000 teams across six cancer types in England showed that 30% of MDTs did not have even written protocols for patient management [24] and there was considerable variability in performance.

Some clinicians [28] have raised concerns about the way cancer MDMs are conducted in UK as frantic business meetings during which there is little scope for learning and educational opportunities for the trainees. Furthermore concerns are raised about their adverse impact on the critical appraisal skills and independent thought of trainees who play a passive role in these meetings. Concerns are also raised about the diminishing role of patients and their preferences and views may not be fully represented in these meetings. These is also a danger of MDM recommendations being conveyed to patients in an authoritarian manner in which consultations are simply used to obtain consent to follow MDM recommendations without allowing patients the ability to fully explore all their available options.

The interfaces between cancer MDTs and primary care physicians are crucial for the continuity of the care, however the specific pathways and methods for handling those interfaces are not well established [1]. 

The scale and the extent of the described practical challenges would vary significantly across different organisations and across different national healthcare systems. No single strategy would be sufficient to address all the challenges described earlier and given the complex nature of cancer MDMs, a multipronged approach would achieve greater overall benefits. 

 In the next section, we explore a novel strategy of using clinical decision support (CDS) technology to address some of the challenges. We describe one such decision support system, developed with an aim to support breast cancer MDT meetings in the UK hospital, to provide concrete examples of the capabilities of advanced CDS systems.

## 6. The Potential of Clinical Decision Support Technology in Cancer MDT Meetings

**Figure 1:**
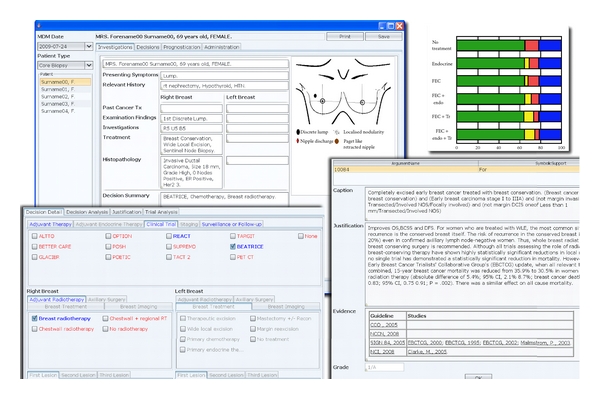
Composite screenshot describing some of the functionalities of an example CDS tool developed for breast cancer MDT meeting. Upper left: the summary screen for the patient. Upper right: one of the many prognostication tools available, Lower left: decision panel where system recommendations and eligible clinical trials are highlighted in blue. Lower right: the evidential justification for each recommended option.

Clinical decision support systems can be defined as ‘‘systems that are designed to be a direct aid to clinical decision-making in which the characteristics of an individual patient are matched to a computerised clinical knowledge base, and patient-specific assessments or recommendations are then presented to the clinician(s) and/or the patient for a decision” [29]. The health information technology such as electronic patient records (EPRs) could assist in structural and administrative aspects of cancer MDTs such as preparation, data collection, presentation, and consistent documentation of decisions. However, advanced CDS systems could offer services well beyond the use of clinical databases and EPRs by actively supporting patient-centred, evidence-based decision-making. One such advanced CDS system called MATE, Multidisciplinary team Assistant and Treatments Elector, is being developed for breast MDT meetings and is being trialed at the London Royal Free hospital. Figure 1 is a composite image of some of the functionalities of MATE.

An advanced CDS system is able to evaluate all available patient data in real time, including comorbidities, and offer prompts, reminders, and suggestions for management in a transparent way. A CDS system can use national guidelines and other high-quality evidence to generate patient-specific recommendations and linking them to the source of evidence for transparency. 

It is essential to emphasise that CDS systems normally only *suggest* optimal management strategy, laying out the medical logic and relevant supporting documentation and research; the decision is of course the responsibility of the members of MDT. Since such systems can compare all MDT decisions with recommendations, it can also be used to carry out prospective audit of MDM decisions. Furthermore CDS technology could also allow clinicians to record their views on guideline recommendations, which can be captured into a hospital or national database for quality audits and informing the ongoing guideline development and update process [30]. Eligibility of patients for recruitment into ongoing trials could also be screened in real time during the MDT meetings. The trainee doctors can run cases through the CDS system and study the recommendations and evidence against their own decisions. 

After the MDM recommendations are discussed with the patients in results clinic, a CDS system could be accessed by patients if they wish to revisit the information about their management pathways. A patient-friendly module of a CDS system can provide patients with access to and explanations of clinical recommendations in an appropriate form, thus helping them to understand the reasons why treatments are being offered and make better informed decisions. Similarly primary care physicians can access the MDT plans and recommendations for their patients to provide appropriate surveillance, survivorship, or palliative care.

MATE provides an example of advanced CDS systems but it should be borne in mind that there is much work to be done to establish the best approach in providing CDS services. Our purpose here is to argue for further research and debate around this important topic, not to assert the clinical benefits of using these technologies in the cancer MDTs.

## 7. Concluding Remarks

Cancer conferences have come a long way in the last 50 years, from a forum for presenting interesting cases to a platform for collaborative day-to-day management of cancer patients. Given the complex nature of cancer MDT meetings, which pose significant difficulties for evaluation, the paucity of high-quality evidence for their effectiveness is not surprising. Significant challenges remain in getting maximum leverage from this important decision-making forum. New research should be directed to investigate better methods to support these heavily loaded but key care planning meetings. An advanced decision support technologies show considerable promise for supporting clinical, operational, and governance aspects of the cancer MDTs with reliability, transparency, and accountability.

## 8. Notes

We performed a detailed literature search for published articles in English for the period 1970 to November 2010. Data were identified by searches of MEDLINE, EMBASE, CINAHL and COCHRANE databases. References of the retrieved articles were also screened. Combinations of search terms ‘‘multidisciplinary”, ‘‘multidisciplinary team”, ‘‘multidisciplinary clinic”, “multidisciplinary cancer conference”, ‘‘multidisciplinary meeting”, ‘‘tumour board” and “cancer” were used. Websites of government agencies and national health care organisations were also searched for relevant documents and reports.

##  Conflict of Interests

The authors declare that they have no conflict of interests.

## Figures and Tables

**Table 1:** Summary of empirical evidence on the effectiveness of cancer MDT meetings.

Outcomes assessed	Study	E*	Total cases	Cancer type	Difference in MDT meeting arm and control arm with respect to the outcome
Survival	[7]	2b	88	Lung	NSD
	[8]	3b	67	Glioma	NSD (18.7 versus 11.9 months, *P* = 0.11)
	[9]	4	240	Lung	NSD
	[10]	4	144	Oesophageal	5 years (52% versus 10%, *P* < 0.001)
	[11]	4	243	Lung	Median (6.6 months versus 3.2 months)^§^
	[12]	4	533	Ovarian	In favour of MDT group^§^
	[13]	4	16035	All cancers	5 years (71% versus 63%, *P* < 0.001)
	[14]	4	—	Lung	1 year (23.5% versus 18.3%)^§^

Quality of life	[7]	2b	88	Lung	NSD

Patient experience	[7]	2b	88	Lung	Improved in MDT group, *P* = 0.01
	[15]	4	269	Breast	Improved in MDT group, *P* < 0.001

Rate of intervention	[11]	4	243	Lung	Patients receiving chemo (23% versus 7%)^§^
	[16]	4	112	Lung	30% ↑ in resection in favour of MDT
	[8]	3b	67	Glioma	Patients having chemo (55% versus 17%)^§^
	[9]	4	240	Lung	↑ in resection (23.4 % versus 12.2%)^§^
	[17]	3b	2935	Colorectal	↑ in trial recruitment (10.3 versus 5.1%)^§^

Time to intervention	[15]	4	269	Breast	Time to treatment (29.6 versus 42.2 days)^§^
	[16]	4	112	Lung	NSD
	[8]	3b	67	Glioma	NSD

Staging accuracy	[18]	3b	118	Upper GI	MDT improved staging accuracy^§^
Costs per patients	[19]	4	208	Melanoma	MDT saved $1600 per patient
Decision quality as prediction of accuracy	[20]	4	50	Lung	NSD, Team discussion did not improve the quality of decision making overall.
Psychological morbidity of team members	[21]	5	72	Breast	lower prevalence of psychiatric morbidity (15.7% versus 26.6% *P* < 0.005)

E*: levels of evidence as defined by Oxford Centre for Evidence-Based Medicine (1a: systematic review of RCTs, 1b: individual RCT (with narrow Confidence Interval), 1c: all or none, 2a: systematic review of cohort studies, 2b: individual cohort study (including low quality RCT), 2c: “Outcomes” Research, 3a: systematic review of case-control studies, 3b: individual Case-Control Study, 4: case-series (and poor quality cohort and case-control studies), 5: expert opinion without explicit critical appraisal, or based on physiology, bench research or “first principles”), NSD: no significant difference found in both groups, ^§^statistically significant differences, and chemo: chemotherapy.

**Table 2:** Challenges in realising the full potential of cancer MDT meeting.

Establishing robust mechanisms for prospective assessment of MDT performance
Ensuring MDT recommendations are followed in the practice
Ensuring adherence with standards including evidence-based guidelines
Establishing reliable interfaces with primary care to ensure continuity of care
Ensuring active patient participation
Achieving right balance of educational and care delivery objectives of this forum
Ensuring the consistent collection of crucial data such as disease staging and outcomes
Limiting exposure of the MDT members to medicolegal liability
